# Exosomes Released by Cerebrolysin-Treated Cerebral Endothelial Cells Reverse Fibrin- or tPA-Impaired Endothelial Cell Permeability

**DOI:** 10.3390/cells15100934

**Published:** 2026-05-19

**Authors:** Hua Teng, Chao Li, Mingjin Wang, Jing Zhang, Yi Zhang, Michael Chopp, Zheng Gang Zhang

**Affiliations:** 1 Department of Neurology, Henry Ford Health, Detroit, MI 48202, USA; 2 Department of Physics, Oakland University, Rochester, MI 48309, USA

**Keywords:** exosomes, Cerebrolysin, cerebral endothelial cells, endothelial integrity, endothelial proteins

## Abstract

Cerebrolysin has a salutary effect on impaired cerebral endothelial cell (CEC) permeability. Using an in vitro endothelial permeability assay, the present study tested the hypothesis that exosomes released by Cerebrolysin-treated CECs (Cerebro-Exos) have a robust therapeutic effect on dysfunctional CECs. Stoichiometric analysis showed marked differences in cargo profiles between Cerebro-Exos and exosomes derived from CECs without Cerebrolysin treatment (Naïve-Exos), in which Cerebro-Exos were highly enriched with metabolic and tight junction related proteins compared to Naïve-Exos. Cerebro-Exos had a superior effect compared to Naïve-Exos on restoring CEC integrity impaired by fibrin and tissue plasminogen activator (tPA). Treatment of fibrin- and tPA-challenged CECs with Cerebro-Exos robustly reduced fibrin- and tPA-augmented proteins involved in inflammation and coagulation and substantially increased fibrin- and tPA-decreased proteins that are related to tight junctions and metabolism. Collectively, these data indicate that Cerebro-Exos have a broad effect on improvement of dysfunctional CECs, which is likely achieved by the alteration of CEC proteins.

## 1. Introduction

Cerebral endothelial cells play an essential role in blood–brain barrier (BBB) and neurovascular homeostasis and in pathological conditions including stroke, traumatic brain injury (TBI), and dementia [1,2,3]. Emerging data indicate that cerebral endothelial cells actively communicate with cells in the neurovascular unit by releasing exosomes [4,5]. Exosomes are endosome-derived nanovesicles that mediate recipient cell biological function by transferring cargo proteins, lipids, and genomic materials to recipient cells [5]. We demonstrated that treating acute stroke in rats with exosomes generated by healthy cerebral endothelial cells improves neurological outcomes and augments the integrity of cerebral microvessels [6].

Cerebrolysin is a neuropeptide preparation that mimics the action of endogenous neurotrophic factors in the brain [7,8]. The therapeutic effects of Cerebrolysin have been demonstrated in clinical and preclinical studies of stroke, TBI, and dementia. For example, treatment of rats subjected to stroke or TBI with Cerebrolysin robustly improves neurological outcomes [9,10,11]. Clinical trials have also shown that Cerebrolysin improves cognitive and functional outcomes in patients with vascular dementia, stroke, or TBI [12,13,14]. Using an in vitro model of human cerebral endothelial permeability, we have demonstrated that Cerebrolysin robustly reduces fibrin- and tissue plasminogen activator (tPA)-induced endothelial cell leakage by substantially decreasing proinflammatory and procoagulant proteins and by increasing the expression of tight junction proteins [3]. These in vitro data provide evidence that Cerebrolysin improves cerebral endothelial cell function.

Exosomes, small extracellular vesicles, mediate intercellular communication by delivering their cargo to recipient cells, consequently leading to changes in recipient cell biological function [5,15]. It is well established that the status of the parent cell impacts the content of its exosomal cargo [16]. For example, exosomes derived from healthy cerebral endothelial cells suppress cerebral vascular inflammation and coagulation after stroke [6]. However, it remains unknown whether treatment of healthy cerebral endothelial cells with Cerebrolysin changes the endothelial cell-released exosome cargo and whether exosomes released by Cerebrolysin-treated endothelial cells affect dysfunctional cerebral endothelial cells. In the present study, we tested the hypothesis that exosomes derived from cerebral endothelial cells treated with Cerebrolysin have a beneficial effect on impaired cerebral endothelial integrity. This study provides insight into molecular mechanisms underlying the therapeutic benefits of Cerebrolysin on cerebral vasculature.

## 2. Materials and Methods

Cerebrolysin was obtained from EVER Pharma (EVER Pharma, Unterach, Austria). Primary human brain microvascular endothelial cells (P3) were purchased from ScienCell (ScienCell, Carlsbad, CA, USA, Cat#: 1000)All cells were CD31-positive as assayed by immunocytochemistry, indicating endothelial cell purity (ScienCell). The cerebral endothelial cells (CECs) were cultured with endothelial cell culture medium (ScienCell, Carlsbad, CA, USA, Cat#: 1001). CECs with four passages were employed in the present study.

### 2.1. Isolation and Characterization of Exosomes

Exosomes isolated from CECs treated with Cerebrolysin or with PBS are referred to as Cerebro-Exos or Naïve-Exos, respectively. Briefly, CECs were treated with Cerebrolysin at a dose of 20 μL/mL for 24 h. After that, the cultured medium was removed and the treated CECs were cultured in fresh exosome-free medium (Cell Systems, Kirland, WA, USA, Cat #: SF-4Z0-500) without Cerebrolysin for 48 h. Exosomes were then isolated from the collected supernatant using a differential ultracentrifugation method according to our published protocol [6]. Briefly, the supernatant was centrifuged at 10,000× *g* for 30 min to remove debris, followed by a second centrifugation at 100,000× *g* for 120 min to obtain a pellet. The isolated exosomes were characterized by nanoparticle tracking analysis (NTA, NS300, Malvern Panalytical, Westborough, MA, USA), transmission electron microscopy (TEM), and Western blotting using antibodies against exosomal markers including CD9 and CD63. The endoplasmic reticulum marker calnexin was used as an exosomal negative quality control.

### 2.2. Cerebral Endothelial Cell Permeability Assay

CEC permeability was measured using published protocols [3,17]. Briefly, a transwell device (0.4 um pore size, Costar, Corning Incorporated, Kennebunk, ME, USA. Cat #: 3413) was used by seeding the CECs at 5 × 10^4^/well in the insert of the transwell for 5 days. Fluorescent-conjugated dextran (FITC–dextran 70 kDa, 0.5 mg/mL, Thermo Fischer Scientific, Waltham, MA, USA, Cat#: D1830) was added to the inserted chamber (the upper well) of the transwell for 30 min. Fluorescent signals at the outer chamber (the bottom well) were measured at wavelengths of 595 nm and 615 nm using a plate reader (Packard, Packard Bioscience Company, Meriden, CT, USA). The trans-endothelial permeability was calculated as (OD_30min_ − OD_0min_) experimental/(OD_30min_ − OD_0min_) control × 100% [18].

### 2.3. The Effects of Naïve-Exos and Cerebro-Exos on Fibrin- or tPA-Impaired CEC Permeability

We previously demonstrated that Cerebrolysin reduces fibrin- or tPA-augmented cell permeability [3]. To examine the effects of Naïve-Exos and Cerebro-Exos on fibrin- or tPA-impaired cell permeability, CECs (5 × 10^4^/well) were cultured in the transwell insert for monolayer formation. The CECs were then treated with tPA at a concentration of 10 µg/mL or fibrin (Sigma-Aldrich, St Louis, MO, USA, Cat#: F3879) at a concentration of 1.5 µg/mL in the presence or absence of exosomes (1 × 10^8^ particles/mL) for an additional 24 h. FITC–dextran was then added to the upper well, and the intensity of FITC signal in the bottom after 30 min was quantified at wavelengths of 595 nm and 615 nm using a fluorescent plate reader as an index of CEC leakage.

### 2.4. Western Blot

CECs were lysed using Pierce RIPA buffer (Thermo Fisher Scientific, Waltham, MA, USA, Cat#: 89901) supplemented with a protease inhibitor cocktail (Calbiochem, Millipore Sigma, Burlington, MA, USA, Cat #: 539131). The lysates were centrifuged at 12,000× *g* at 4 °C to remove cell debris, and supernatants were collected as total protein extracts. For exosomal protein isolation, the purified exosomes were directly lysed in RIPA buffer under the same conditions. Protein concentration was determined using a BCA kit (Thermo Fisher Scientific, Waltham, MA, USA, Cat#: 23227). Equal amounts of total protein (20 µg for CEC lysates and 6 µg for exosome lysates) were resolved on NuPAGE 10% Bis-Tris gels (Invitrogen, Thermo Fisher Scientific, Waltham, MA, USA, Cat#: NP0301BOX) and subsequently transferred onto PVDF membranes using a Trans-Blot Turbo transfer system (Bio-Rad, Hercules, CA, USA). Membranes were blocked and incubated with primary antibodies: rabbit polyclonal antibody anti-CD9 (ab223052, Abcam, Waltham, MA, USA), mouse monoclonal anti-CD63 (SC-5275, Santa Cruz Biotechnology, Santa Cruz, CA, USA), rat monoclonal anti-calnexin (699401, Biolegend, San Diego, CA, USA), mouse monoclonal antibody against intercellular adhesion molecule 1 (ICAM1, ab171123, Abcam, Waltham, MA, USA), rabbit polyclonal antibody against high mobility group box 1 (HMGB1, ab79823, Abcam, Waltham, MA, USA), rabbit polyclonal antibodies against tumor necrosis factor α (TNFα, 3707, Cell Signaling Technology, Danvers, MA, USA), rabbit monoclonal antibody against phosphorylated NFκB-p65 (NFκB-p65, 8242, Cell Signaling Technology, Danvers, MA, USA), rabbit polyclonal antibodies against zonular 1 (ZO1, 5406, Cell Signaling Technology, Danvers, MA, USA), rabbit polyclonal antibodies against occludin (ab31721, Abcam, Waltham, MA, USA), rabbit polyclonal antibodies against claudin 5 (ab15106, Abcam, Waltham, MA, USA), and mouse monoclonal antibody against β-actin (ab8226, Abcam, Waltham, MA, USA). Horseradish peroxidase-conjugated secondary antibodies were used. β-actin was used as a loading control. The intensity of individual bands on Western blots was normalized to corresponding β-actin bands and quantified with ImageJ software (fiji win64).

### 2.5. Proteomic and Bioinformatic Analysis

To investigate the cargo profiles of proteins in Cerebro-Exos and Naïve-Exos, as well as in cells treated with Cerebro-Exos and Naïve-Exos, total proteins were extracted from these exosomes and cells. The proteins in exosomes and cells were measured using label-free quantitative Q-Exactive mass spectrometry. Following protein identification, exclusive spectrum counts were utilized for relative protein quantification. To address zero-value dropouts in the spectral data, a standard pseudocount of 0.1 was added to all samples prior to base-2 logarithmic (log_2_) transformation.

Differentially expressed proteins (DEPs) between experimental groups were identified utilizing a stringent dual-filter criterion to ensure robust biological relevance: statistical significance was determined via an independent-sample *t*-test (*p* < 0.05), and effective size was defined by an absolute fold change >1.0 (corresponding to a >2.0-fold or <0.5-fold change in raw expression). To interpret the functional consequences of these proteomic alterations, targeted pathway enrichment analysis was performed. Fisher’s exact test was used to identify significantly overrepresented biological pathways (e.g., mitochondrial metabolism, cytoskeletal dynamics, and inflammatory signaling) among the DEPs relative to the background proteome. Global statistical patterns were visualized using volcano plots. To assess expression consistency across biological replicates, functionally grouped heatmaps were generated. Spectral counts were row-wise Z-score normalized, grouped by functional pathway, and ordered within each group by descending fold-change magnitude.

### 2.6. Statistical Analysis

All continuous data are presented as means ± standard deviation (SD). Data normality was assessed using the Shapiro–Wilk test. For comparisons involving more than two groups, statistical significance was determined using one-way analysis of variance (ANOVA) followed by Tukey’s post hoc test, performed with GraphPad Prism software (version 10.0.3, GraphPad Software Inc., Boston, MA, USA).

## 3. Results

### 3.1. CEC Culture and Characterization of Cerebro-Exos

**Figure 1:**
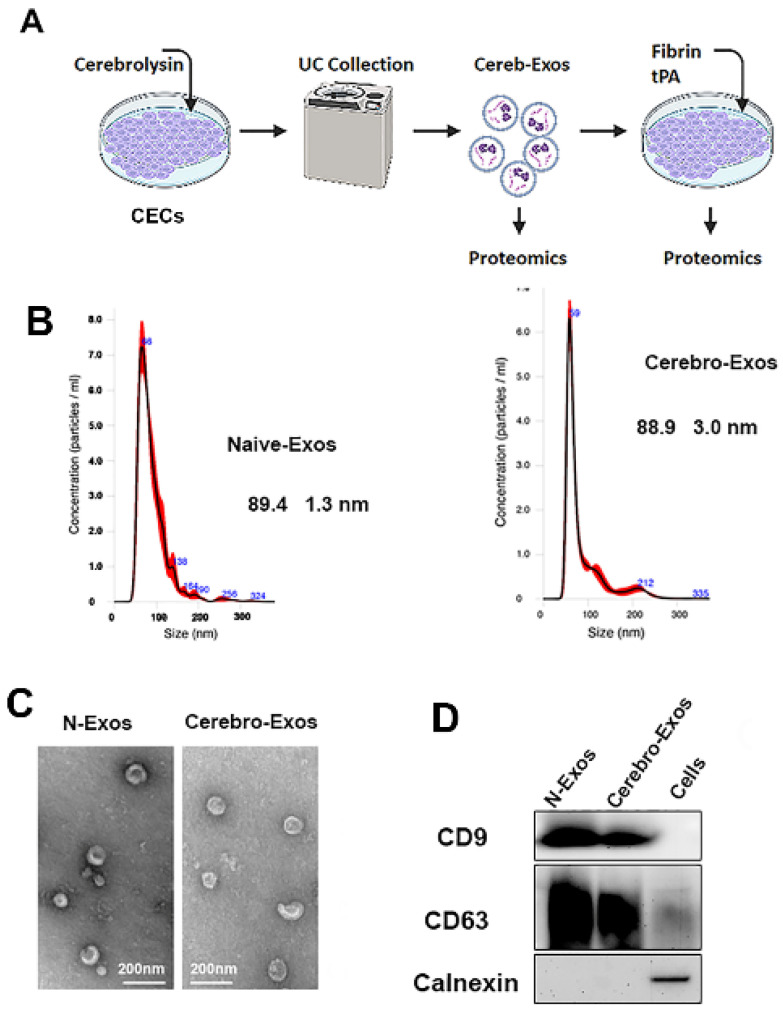
Characterizations of Naïve-Exos and Cerebro-Exos. (**A**) Schematic illustration of exosome isolation. (**B**) Size distribution of Naïve- and Cerebro-Exos determined by nanoparticle tracking analysis (NTA). (**C**) Representative transmission electron microscopy (TEM) showing exosome morphology. (**D**) Western blots (blot) analysis of exosomal markers CD9 and CD63 with calnexin used as a negative control.

We previously demonstrated that healthy CECs produce exosomes [6]. To examine whether Cerebrolysin treatment affects the characteristics of endothelial exosomes, CECs were treated with Cerebrolysin or PBS. After the treatments, CECs were cultured in fresh exosome-free endothelial cell medium without Cerebrolysin. Exosomes were isolated from supernatant collected from the cultured CECs using differential ultracentrifugation (Figure 1A) [6]. Nanoparticle tracking analysis (NTA) revealed that particles isolated from Cerebrolysin (Cerebro-Exos) or PBS (Naïve-Exos) had a mean size of 88.9 ± 3.0 nm or 89.4 ± 1.3 nm, respectively, not significantly different between the two exosome groups (Figure 1B). Transmission electron microscopy (TEM) analysis showed that the particles had typical exosomal morphology, and Western blots showed that Cerebro-Exos and Naïve-Exos contained the exosomal marker proteins CD9 and CD63 (Figure 1C) and not the endoplasmic protein calnexin (Figure 1D). These data indicate that Cerebrolysin treatment does not change the structure of endothelial cell-generated exosomes compared to naïve CEC-released exosomes.

### 3.2. Cerebro-Exos Reduce Fibrin- and tPA-Impaired Endothelial Cell Barrier Integrity and Decrease Proteins Related to Impaired Barrier Integrity

**Figure 2:**
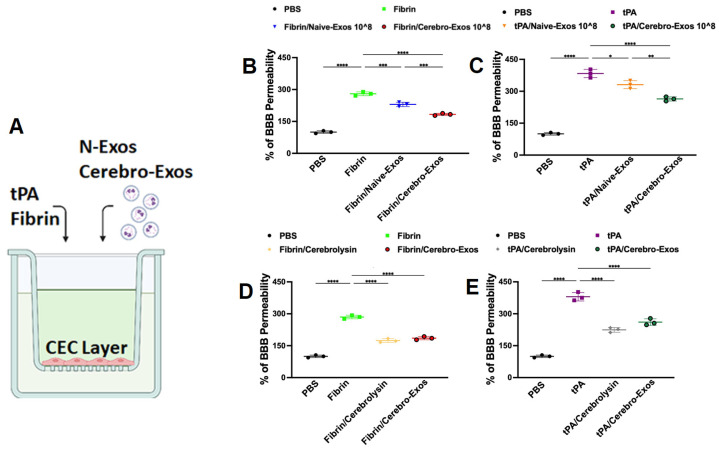
Effects of Naïve-Exos, Cerebro-Exos, and Cerebrolysin on fibrin- or tPA-impaired CEC permeability. (**A**) Schematic of the transwell permeability assay, showing CECs seeded on the insert. Trans-endothelial permeability was quantified by measuring fluorescent dextran in the lower chamber. (**B**,**C**) Quantification of permeability in CECs stimulated with fibrin (**B**) or tPA (**C**) with or without treatment with Naïve- (N-Exos) and Cerebro-Exos (C-Exos) at 1 × 10^8^ particles/mL. (**D**,**E**) Effects of Cerebrolysin and Cerebro-Exos on fibrin-induced (**D**) or tPA-induced (**E**) permeability. Data are presented as means ± SD. Statistical significance was determined by one-way analysis of variance (ANOVA) followed by Tukey’s post hoc test (*n* = 3 per group). *, **, ***, **** = *p* < 0.05, 0.01, 0.001, 0.0001, respectively.

Using an in vitro endothelial cell permeability assay, we examined the effects of Cerebro-Exos and Naïve-Exos on healthy CEC permeability (Figure 2A). Neither Cerebro-Exos nor Naïve-Exos significantly changed endothelial permeability at doses of 1 × 10^7^ particles/mL or 1 × 10^8^ particles/mL. We previously demonstrated that healthy CECs challenged by fibrin and tPA exhibit increased permeability, while Cerebrolysin robustly reduces fibrin- and tPA-augmented permeability [3]. We thus examined whether Cerebro-Exos affect fibrin- or tPA-exacerbated CEC permeability as does Cerebrolysin. Consistent with our previous data, treatment of healthy CECs with fibrin or tPA robustly increased CEC permeability, which was significantly reduced by Cerebrolysin (Figure 2B,C). The effect of Cerebro-Exos on fibrin- or tPA-challenged CEC permeability was dose dependent, with a more robust impact at 1 × 10^8^ particles/mL (Figure 2B,C and Supplement Figure S1). Accordingly, a dose of Cerebro-Exos at 1 × 10^8^ particles/mL was employed in all the following experiments. Compared to Naïve-Exos, Cerebro-Exos significantly (*p* < 0.01) decreased fibrin- or tPA-induced permeability, although Naïve-Exos substantially reduced the fibrin- or tPA-increased permeability, as we previously demonstrated (Figure 2B,C) [6]. Importantly, Cerebro-Exos significantly (*p* < 0.01) reduced the fibrin- and tPA-augmented CEC permeability to a level that was comparable to Cerebrolysin treatment (Figure 2D,E). These data suggest that Cerebro-Exos have a potent functional effect on restoring impaired endothelial barrier integrity.

**Figure 3:**
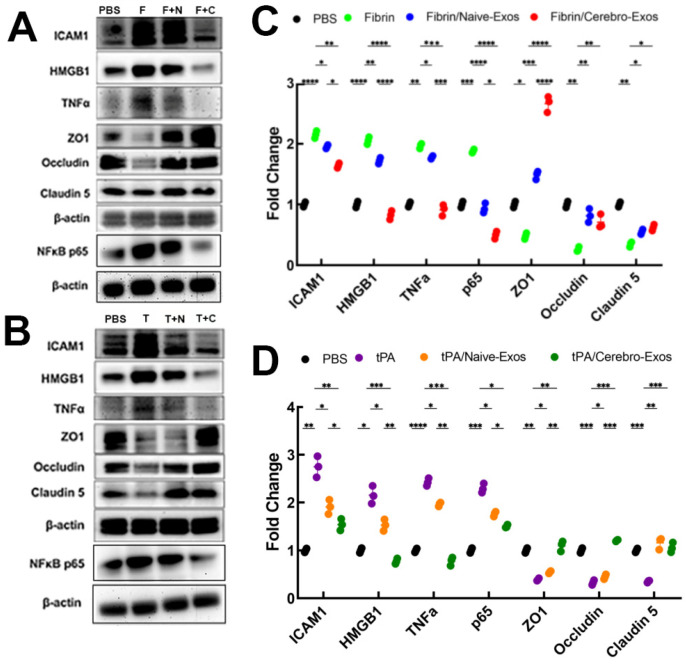
Effects of Naïve-Exos and Cerebro-Exos on cerebral endothelial cell proteins altered by fibrin and tPA. (**A**,**B**) Representative Western blots showing protein expression in CECs following fibrin (**A**) or tPA (**B**) stimulation with and without Naïve-Exos (N-Exos) and Cerebro-Exos (C-Exos) treatments. (**C**,**D**) Quantification of protein expression corresponding to (**A**) and (**B**), respectively. β-actin was used as a loading control. Statistical significance was determined using one-way analysis of variance (ANOVA) followed by Tukey’s post hoc test (*n* = 3 per group). *, **, ***, **** = *p* < 0.05, 0.01, 0.001, 0.0001, respectively. F = fibrin, F + N = fibrin + Naïve-Exos, F + C = fibrin + Cerebro-Exos, T = tPA, T + N = tPA + Naïve-Exos, and T + C = tPA + Cerebro-Exos.

We previously demonstrated that in addition to cell permeability, Cerebrolysin reduces the expression of fibrin- and tPA-triggered inflammatory and coagulation proteins [3]. We thus examined the effect of Cerebro-Exos on these proteins. Fibrin and tPA significantly (*p* < 0.01) increased proinflammatory proteins and decreased tight junction proteins in CECs, respectively (Figure 3A,B), whereas both Naïve-Exos and Cerebro-Exos robustly reduced fibrin- and tPA-augmented CEC proteins and increased their reduced tight junction proteins (Figure 3A,B). Compared to Naïve-Exos, Cerebro-Exos further significantly (*p* < 0.01) altered the fibrin- and tPA-changed proteins (Figure 3A,B). These data indicate that Cerebro-Exos have more potent effects than Naïve-Exos on reduction in fibrin- and tPA-triggered proinflammatory and procoagulant proteins and on augmentation of tight junction proteins.

### 3.3. Cerebro-Exo Protein Cargo Has a Distinct Profile from Naïve-Exo Protein Cargo

**Figure 4:**
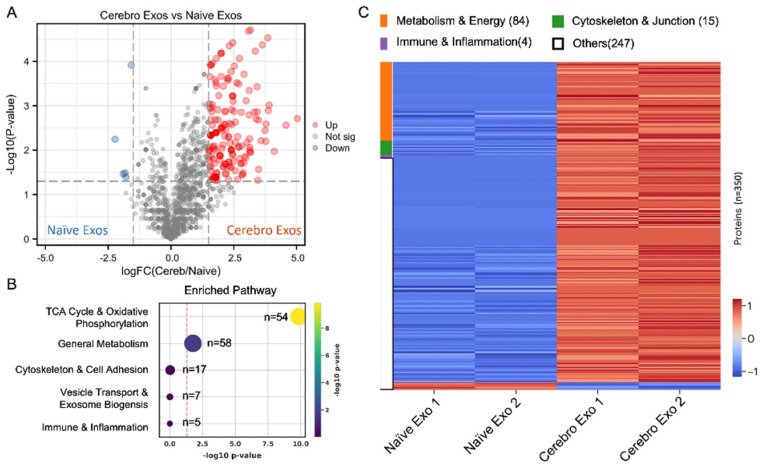
Naïve-Exo and Cerebro-Exo protein cargo profiles. (**A**) Volcano plot comparing exosome proteomes between Naïve-Exos and Cerebro-Exos, identifying 350 high-confidence differentiate expression proteins. (**B**) Pathway enrichment analysis (Fisher’s exact test) showing pathway enriched in Cerebro-Exos. Numbers indicate protein counts per pathway. (**C**) Partial hierarchical clustering heatmap of relative protein abundance across Naïve-Exos and Cerebro-Exos samples, with two replicates per group.

Since exosome cargo plays critical roles in mediating recipient cell function [5,19], proteomic analysis was performed to identify potential candidate proteins within Cerebro-Exo cargo that could have underlain the observed changes in permeability. Using label-free quantitative LC–MS, we thus analyzed global cargo protein profiles of Cerebro-Exos and Naïve-Exos. We identified 1942 proteins from the spectral counts. To define the most robust biological differences, DEP analysis was performed using a stringent dual-filter criterion (absolute log_2_ fold change > 1.0 [>2-fold change] and independent-sample *t*-test, *p* < 0.05). This approach identified 350 high-confidence DEPs, of which 342 proteins were significantly enriched in Cerebro-Exos and 8 in Naïve-Exos. A volcano plot confirmed this pronounced and asymmetrical enrichment of cargo within Cerebro-Exos (Figure 4A, Supplement Data S1). To interpret the functional consequences of this 350-DEP signature, pathway enrichment analysis was performed using Fisher’s exact test. The results demonstrated that Cerebro-Exos were highly enriched in proteins regulating core metabolic and structural processes. Notably, the tricarboxylic acid (TCA) cycle and oxidative phosphorylation (*n* = 54 proteins, *p* < 0.001) represented the most significantly enriched pathways, followed by ribosome/protein synthesis and cytoskeleton/cell adhesion networks (Figure 4B). A functionally grouped heatmap further confirmed the consistency of these proteomic shifts across biological replicates (Figure 4C), clearly segregating exosome populations and demonstrating coordinated upregulation of metabolic machinery, cell adhesion components, and anti-inflammatory regulators in Cerebro-Exos compared to Naïve-Exos.

### 3.4. Cerebro-Exos Alter Fibrin- and tPA-Increased Protein Profiles That Are Associated with CEC Barrier Impairment

**Figure 5:**
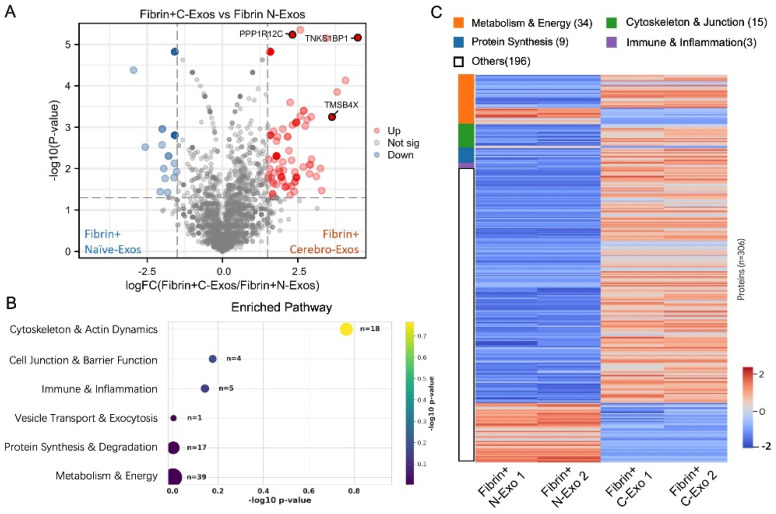
Effects of Naïve-Exos and Cerebro-Exos on cerebral endothelial proteome altered by fibrin. (**A**) Volcano plot comparing proteomes of fibrin-stimulated CECs treated with Naïve-Exos (Fibrin + Naïve-Exos) versus Cerebro-Exos (Fibrin + Cerebro-Exos), identifying 306 high-confidence differentially expressed proteins. (**B**) Pathway enrichment analysis highlighting pathways enriched in CECs treated with fibrin and Cerebro-Exos. Numbers indicate protein counts per pathway. (**C**) Hierarchical clustering heatmap of relative protein abundance across treatment groups, with two replicates per group.

To evaluate the salutary effect of Cerebro-Exos on fibrin- and tPA-impaired endothelial cell permeability, proteomic profiling was performed in CECs across four conditions: saline (control), fibrin alone, fibrin + Naïve-Exos, and fibrin + Cerebro-Exos. To determine Cerebro-Exo-specific effects on CECs, DEP analysis was conducted comparing fibrin + Cerebro-Exos versus fibrin + Naïve-Exos using the same stringent criteria (absolute log_2_ fold change > 1.0, *p* < 0.05). This analysis identified 306 significant DEPs—244 upregulated and 62 downregulated proteins—following Cerebro-Exo treatment of fibrin-exposed CECs, indicating substantial alterations in the fibrin-stimulated cellular proteome (Figure 5A; Supplementary Data S2). Among the abundant proteins were PPP1R12C (protein phosphatase 1 regulatory subunit 12C), which regulates myosin light-chain phosphorylation [20,21], TMSB4X (thymosin beta 4, X-linked), which promotes angiogenesis [22], and TNKS1BP1 (tankyrase 1-binding protein 1), which is involved in Wnt/β-catenin signaling [23]. Pathway enrichment analysis revealed that the Cerebro-Exo treatment primarily impacted structural integrity. The most significantly enriched pathways included cytoskeleton and actin dynamics (*n* = 18), protein synthesis and degradation (*n* = 17), and cell junction/barrier function (*n* = 4, Figure 5B). A functionally grouped heatmap across four individual samples of fibrin-stimulated CECs treated with Cerebro-Exos and Naïve-Exos (Figure 5C) demonstrated that Cerebro-Exo treatment of fibrin-exposed CECs had a greater impact on metabolic and cytoskeletal proteins in these CECs than treatment with Naïve-Exos.

**Figure 6:**
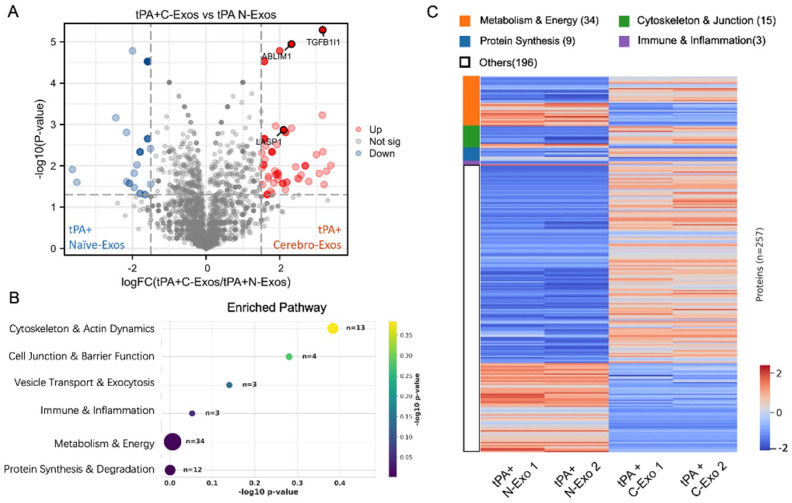
Effects of Naïve-Exos and Cerebro-Exos on cerebral endothelial proteome altered by tPA. (**A**) Volcano plot comparing CEC proteomes from tPA-stimulated CECs treated with Naïve-Exos (tPA + Naïve-Exos) and Cerebro-Exos (tPA + Cerebro-Exos), identifying 257 high-confidence differentially expressed proteins. (**B**) Pathway enrichment analysis highlighting pathways enriched in CECs treated with tPA and Cerebro-Exos. Numbers indicate protein counts per pathway. (**C**) Hierarchical clustering heatmap of relative protein abundance across treatment groups, with two replicates per group.

Next, we examined tPA-stimulated CECs using the same experimental framework. DEP analysis identified 257 significant proteins distinguishing Cerebro-Exo and Naïve-Exo treatments, with 173 proteins increased in the Cerebro-Exo group (Figure 6A). Increased proteins included TGFB1I1 (Hic-5), which mediates TGFβ-driven endothelial contractility [24], LASP1 (LIM and SH3 protein 1), which regulates cell migration [25], and ABLIM1 (actin-binding LIM protein 1), which regulates cell junction stability [26]. Pathway enrichment analysis revealed that these proteins are involved in pathways in cytoskeleton and actin dynamics (*n* = 13) and cell junction function (*n* = 4, Figure 6B). Heatmap visualization (Figure 6C) showed that compared to the Naïve-Exo treatment, the Cerebro-Exo treatment induced more CEC proteins that regulate cytoskeleton dynamics and cell junction function.

## 4. Discussion

Cerebrolysin is clinically used for treatment of stroke and other neurological disorders [12,13,14]. The present in vitro study demonstrated for the first time that Cerebro-Exos reduce fibrin- and tPA-induced CEC permeability, achieving an effect comparable to that of the parent agent Cerebrolysin. Importantly, our findings further provide potential mechanistic insights into how Cerebro-Exos confer protective effects on dysfunctional CECs, thereby extending the therapeutic paradigm of Cerebrolysin beyond its direct pharmacological action.

Both clinical and preclinical studies have consistently shown that Cerebrolysin exerts therapeutic benefits in neurological disorders, including stroke, TBI, and neurodegenerative diseases [9,10,11]. A key mechanism underlying these effects is the restoration of CEC function and blood–brain barrier (BBB) integrity [6]. In parallel, it is well established that endothelial cells, including CECs, constitutively release exosomes that act as critical mediators of intercellular communication within the neurovascular unit. These exosomes regulate vascular homeostasis by transferring bioactive cargo to recipient cells. Our previous work demonstrated that naïve CEC-derived exosomes mitigate fibrin- and tPA-impaired endothelial permeability and reduce BBB leakage in experimental stroke models [6]. However, whether exosomes released by Cerebrolysin-treated CECs possess enhanced functional properties has not been investigated. The present study fills this gap by showing that Cerebro-Exos exert a superior protective effect compared to Naïve-Exos in reducing fibrin- or tPA-induced CEC permeability. This enhanced efficacy is associated with marked suppression of proinflammatory and procoagulant signaling pathways, including reductions in ICAM-1, TNF-α, and NF-κB activation, alongside upregulation of key BBB junctional proteins. These findings suggest that Cerebrolysin not only directly modulates CEC function but also reprograms CEC exosomal signaling to promulgate and amplify vascular protection. Together with prior in vivo evidence of Cerebrolysin-mediated neurovascular recovery, our data support a model in which endogenous exosomes released from Cerebrolysin-treated CECs act in conjunction with Cerebrolysin to promote brain repair and may also influence peripheral organ responses following neurological injury.

Exosomes exert their biological effects primarily through the transfer of proteins, RNAs, and lipids that reprogram recipient cell function [5,19]. In this study, quantitative proteomic analysis revealed distinct cargo profiles between Cerebro-Exos and Naïve-Exos, with Cerebro-Exos showing significant enrichment in proteins associated with cellular metabolism, oxidative phosphorylation, and tight junction regulation. These metabolic adaptations are particularly relevant, as endothelial energy metabolism is increasingly recognized as a key regulator of BBB integrity. Furthermore, proteomic analysis of recipient CECs indicated that Cerebro-Exos modulate several critical pathways governing endothelial barrier function, including actin cytoskeleton organization, actomyosin contractility, and barrier-inductive signaling pathways such as Wnt/β-catenin and TGFβ. These pathways are central regulators of BBB stability [27,28,29]. Mechanistically, specific cargo proteins enriched in Cerebro-Exos appear to converge on these pathways. For example, PPP1R12C (a regulatory subunit of myosin phosphatase) and TGFB1I1 (Hic-5) may coordinately regulate RhoA-dependent cytoskeletal tension and endothelial contractility, a critical determinant of paracellular permeability. TNKS1BP1 is implicated in modulation of Wnt/β-catenin signaling, which is essential for BBB maintenance and endothelial specialization. Additionally, ABLIM1, LASP1, and TMSB4X are cytoskeleton-associated proteins that likely function as a coordinated regulatory module to stabilize actin dynamics and endothelial junctional complexes. Collectively, these data suggest that Cerebro-Exos reinforce BBB integrity through integrated regulation of cytoskeletal remodeling, signaling pathways, and metabolic homeostasis. Disruption of these processes is a well-established driver of BBB dysfunction in neurological diseases, further supporting the broad therapeutic potential of Cerebro-Exos.

Because exosome-mediated cargo delivery is a highly efficient and biologically optimized process, it may offer advantages over conventional pharmacological agents or synthetic nanoparticle systems [5,30]. Notably, exogenously administered CEC-derived exosomes have been shown to cross the BBB and preferentially target neurovascular unit cells in vivo [6], highlighting their potential as a delivery platform. Based on these observations, we speculate that Cerebro-Exos may provide superior targeting efficiency and therapeutic efficacy compared to Cerebrolysin alone, not only within the brain but also in peripheral organs affected by systemic responses to neurological injury. This hypothesis warrants further in in vivo models.

Finally, while the current study focused on proteomic alterations, exosomal RNA and lipid cargo are also likely to contribute significantly to the observed therapeutic effects. Future multi-omic analyses integrating transcriptomics and lipidomics will be essential to fully elucidate the molecular mechanisms underlying Cerebro-Exo-mediated endothelial protection and to identify key therapeutic cargo components.

## Data Availability

The original contributions presented in this study are included in the article/Supplementary Material. Further inquiries can be directed to the corresponding author.

## Supplementary Materials

The following supporting information can be downloaded at https://www.mdpi.com/article/10.3390/cells15100934/s1, Figure S1: A dose response of Naïve-Exos and Cerebro-Exos on fibrin- or tPA-induced cerebral endothelial cell (CEC) per-meability; Supplemental Data S1: Original proteomic data of EV cargo proteins for Figure 3; Supplemental Data S2: Original proteomic data of CECs for Figure 4 and Figure 5; Supplemental Data S3: Original Western blots used for Figure 3.

## Abbreviations

CEC	cerebral endothelial cell
Cerebro-Exos	exosomes released by Cerebrolysin-treated CECs
Naïve-Exos	exosomes derived from CECs without Cerebrolysin treatment
tPA	tissue plasminogen activator
BBB	blood–brain barrier
TBI	traumatic brain injury
ICAM1	intercellular adhesion molecule 1
TNFα	tumor necrosis factor α
