# Supplementary material for: Exosomes Released by Cerebrolysin-Treated Cerebral Endothelial Cells Reverse Fibrin- or tPA-Impaired Endothelial Cell Permeability
Source: Cells. 2026 May 19;15(10):934. doi: 10.3390/cells15100934 (PMC13205112; doi:10.3390/cells15100934)
Supplement: Supplementary file 1 [file cells-15-00934-s001.zip › Supplement data-figure S1.pdf]

Supplement data:

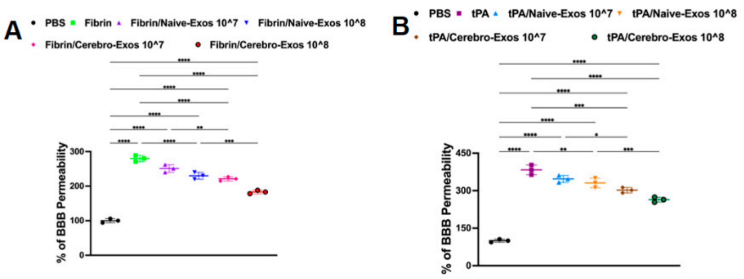

Figure S1: A dose response **of Naïve-Exos and Cerebro-Exos on fibrin- or tPA-induced cerebral endothelial cell (CEC) permeability.** (**A, B**) Quantification of permeability in CECs stimulated with fibrin (**A**) or tPA (**B**) with or without treatment with Naïve-Exos (N-Exos) and Cerebro-Exos (C-Exos) at $1x10^7$ particles/ml or $1x10^8$ particles/ml. (**D, E**) Effects of Cerebrolysin and Cerebro-Exos on fibrin-induced (**D**) or tPA-induced (**E**) permeability. Data are presented as mean ± SD. Statistical significance was determined by one-way analysis of variance (ANOVA) followed by Tukey's post-hoc test (n=3 per group). *, **, ***, **** = p<0.05, 0.01, 0.001, 0.0001, respectively.
